# The ubiquitous sex differences in adolescent mental health: Do we overlook parts of a more complex puzzle?

**DOI:** 10.3389/fpsyg.2025.1707505

**Published:** 2025-12-18

**Authors:** Sara Madeleine Kristensen, Lene Vestad

**Affiliations:** 1 Forebygging.no, Narvik, Norway; 2 Department of Health Promotion and Development, Universitetet i Bergen, Bergen, Norway; 3 Faculty of Arts and Education, Universitetet i Stavanger Laeringsmiljosenteret, Stavanger, Norway

**Keywords:** adolescence, mental health, sex differences, invariance, multiple indicators multiple causes

## Abstract

**Introduction:**

This study investigates sex differences in three widely used mental health instruments: the Warwick-Edinburgh Mental Wellbeing Scale (WEMWBS) and the Hopkins Symptom Checklist (HSCL)-5, and HSCL-10. The psychometric properties and differential item functioning (DIF) of the instruments were explored using measurement invariance tests and multiple indicators multiple causes (MIMIC) models to more fully inspect potential indicator-level differences that may lead to bias.

**Methods:**

There were two adolescent samples in this study. One sample was in grade 8 (ages range: 13–14; 49% females), and the other was in grade 11 (age range: 16–17; 45% females).

**Results:**

The results indicate (1) a dissimilar contribution of some indicators to their latent construct across sexes or a mean sex difference in indicators that are not captured by the construct, and (2) that several indicators of the instruments might be biased regarding sex.

**Discussion:**

This study contributes to our knowledge of the complexity of sex differences in the measurement of adolescent mental health.

## Introduction

Mental health is more than the absence of mental disorders—it is defined as *a state of mental wellbeing that enables people to cope with the stresses of life, realize their abilities, learn well and work well, and contribute to their community* (WHO, 2022). Mental wellbeing is the positive aspect of mental health, and it includes affect (i.e., hedonistic feelings of happiness and life satisfaction: Diener et al., 1985; Huppert et al., 2009) and psychological functioning (i.e., the eudaimonic perspective of living towards a purpose and doing well: Ryan and Deci, 2001; Ryff, 1989; Vittersø et al., 2010). The construct encompasses an individual’s affective-emotional states, cognitive-evaluative dimensions, and psychological functioning (Tennant et al., 2007). In contrast, psychological distress is a negative aspect of mental health. It is a state of mental suffering characterized by symptoms of anxiety (e.g., feeling tense, worrying, and restlessness) and depression (e.g., hopelessness and negative affect) (Strand et al., 2003). Globally, anxiety and depression are the most common conditions of poor mental health (IHME, 2024), and it is estimated that around 2.8 and 4.6 percent of adolescents suffer from depression and anxiety, respectively (WHO, 2021).

Adolescence is a developmental period characterized by extensive physical, social, and psychological changes (Blakemore, 2019). It is a time wherein the prevalence of mental health challenges increases, and it is considered to be among the leading causes of illness and disability (Collishaw, 2015). Indeed, adolescence has been recognized as a crucial period in life to achieve human potential (Patton et al., 2016). In line with this acknowledgment, a call to action has been made to improve adolescent mental health (Weiss and Ferrand, 2019). To support this objective, it is key to measure mental health constructs in adolescent samples accurately (Black et al., 2024; Black et al., 2023). Increasing knowledge of the psychometric properties of mental health measures might improve the accuracy of how we employ instruments in practical settings, such as screening processes, prevalence reporting, and intervention measures. Indeed, because research-generated information is widely used by policymakers, mental health professionals, and other researchers—leading to societal developments and changes in empirical and practical practices—unbiased instruments are paramount for accurately gauging mental health disparities across groups, such as sexes.

An important aspect to consider when investigating the assessment of mental health constructs in adolescence might be the overwhelming evidence of a sex disparity in mental wellbeing and psychological distress. For instance, a recent study found that the gender gap in adolescent mental health is pervasive across cultures, wherein females report experiences of worse mental health than males (Campbell et al., 2021). Further, this gap in adolescence has increased over time (Högberg et al., 2020), suggesting a progressive polarization of mental health across genders (Ross et al., 2017). Globally, mental health problems are a challenge, and among the Nordic countries, Norway has the highest prevalence of psychological distress in the youth population (Ottová-Jordan et al., 2015). Moreover, a recent review and meta-analysis found that the mean score of mental health problems in Norwegian adolescents has increased by 17% among females and 5% among males from 1992 to 2019 (Potrebny et al., 2024). The large gap in mental health gives reason to investigate in more detail how female and male adolescent potentially differ in their perceived mental health.

### Equivalence and potential sex differences in adolescent mental wellbeing and psychological distress

The onset of puberty and identity development are essential aspects of adolescence, shaping how young individuals conform to expected gender roles (Saewyc, 2017). During this time, social pressures increase, and peer friendships become more important, necessitating adjustments to meet social expectations (Blakemore and Mills, 2014). This study adopts a relational theoretical perspective, highlighting how an individual’s environment and context influence the dual process of mental health development (Lerner et al., 2018).

“Mental wellbeing,” as measured by the Warwick–Edinburgh Mental Wellbeing Scale (WEMWBS), encompasses both hedonism (the pursuit of pleasure) and eudaimonia (the pursuit of a flourishing life) (Ryan et al., 2008). These concepts are indicated by factors such as happiness and positive emotions (Huppert et al., 2009) as well as living with purpose and achieving overall wellbeing (Ryff, 1989; Vittersø et al., 2010). When examining wellbeing during adolescence, males report higher average levels than females, although this difference diminishes with age (Gestsdottir et al., 2015). At a more nuanced conceptual level, it is suggested that female adolescents generally report higher levels of eudaimonic wellbeing. At the same time, males tend to emphasize hedonic aspects, such as the importance of feeling good (e.g., Yoon et al., 2023). Supporting this idea, a relational theoretical perspective indicates that the search for purpose and attentiveness to others in relationships are more common wellbeing traits among female adolescents than males (Frydenberg, 2008).

Since WEMWBS is widely used in research and has consistently demonstrated strong factorial measurement invariance across sexes in adolescence (McKay and Andretta, 2017; Oyebode et al., 2023; Smith et al., 2017), this implies that the measure is interpreted similarly for females and males. However, potential differences in wellbeing (Tennant et al., 2007) may exist given the conceptual distinctions (hedonic and eudaimonic). Based on the above, measurement equivalence is expected at the factorial level, yet stronger associations are expected for indicators representing eudaimonic aspects of wellbeing, and the latent construct in early and later adolescence among females. In contrast, a stronger relationship between males and hedonic indicators is anticipated.

Psychological distress encompasses negative affect, adverse arousal, and poor functioning (Schmalbach et al., 2021). Adolescence includes a range of cognitive and social changes, as well as mood fluctuations that can increase vulnerability to psychological distress, crises, or mental health issues (Lahey et al., 2017). The Hopkins Symptom Checklist (HSCL)-5 and HSCL-10 scales both capture symptoms of anxiety and depression (Strand et al., 2003) and are frequently used as a population screening tool in Nordic countries. The scales have been validated in adolescent samples (Clarke et al., 2011; Sirpal et al., 2016) and showed good psychometric properties also in the Norwegian setting (e.g., Smith et al., 2017; Strand et al., 2003).

However, some research implies that some indicators of the HSCL-10 and HSCL-5 might function differently across sexes, a notion that is underscored by past empirical research (e.g., Black et al., 2023). For instance, two Norwegian studies found that the HSCL-10 item 6 ‘Difficulties in falling asleep or staying asleep’ might not appropriately indicate psychological distress in both genders. Kleppang and Hagquist (2016) found that item 6 showed uniform DIF across genders, in which it was uniformly favored by males regardless of their latent variable score. Similarly, Finbråten et al. (2021) found a gender discrepancy in the same item. They argued that these discrepancies might be explained by differences in sleep problems, in which sleep deficiency, social jetlag, and poor sleep quality tend to impact females more than males. In contrast, one study found that both HSCL-10 and HSCL-5 achieved strong measurement invariance across genders in the ages of 14–29 (Schmalbach et al., 2021), indicating no discrepancies between genders on the factor loadings and intercepts of the latent variables.

Hence, past research is inconsistent regarding equivalence across sexes for HCSL and the specific sleep difficulties items. However, the prevalence of psychological distress (symptoms of anxiety and depression) among adolescent females is found to be significantly worse than for males (Potrebny et al., 2024). This may relate to the fact that females report higher levels of interpersonal stressors and typically rely on emotion-focused or avoidant coping strategies than males (Hankin et al., 2007; Lazarus, 2006; Rudolph et al., 2000). Thus far, over twice as many males as females die by suicide each year (WHO, 2024)—often referred to as a gender paradox, wherein although males have fewer suicide attempts, they are more successful in their attempts than females (e.g., Miranda-Mendizabal et al., 2019). One of the major contributors to suicide in males is poor mental health, specifically depression, which seems to be especially challenging to recognize (Barrigon and Cegla-Schvartzman, 2020). The complex pathways of sex differences regarding psychological distress make it unclear whether females consistently exhibit higher levels of anxiety and depression, if this view lacks detailed information, and whether age plays a role in these differences. Taken together, this suggests that expectations regarding sex disparities at the item level are explorative by nature.

### Study aims

This study aims to expand our knowledge of how indicators and latent factors of mental wellbeing and psychological distress function across sexes. Two samples from the Norwegian adolescent population are used to examine whether specific items of selected measures have different measurement properties for females and males, irrespective of group-mean differences of three widely used mental wellbeing and distress instruments. Non-invariance of items across sexes might indicate a dissimilar contribution of indicators to their latent construct or a mean difference in indicators that are not captured by the latent construct, respectively (Putnick and Bornstein, 2016). Further, differentially functioning indicators across sexes might produce measurement bias, leading to incorrect results. It may also help identify specifications for administering measurements separately across groups (Woods et al., 2009).

The main objective in this study is, therefore, to investigate differential item functioning (DIF) employing multiple indicators multiple causes (MIMIC) structural equation models (SEMs) to gain more knowledge of the potential sex differences in three distinct and frequently used instruments: Warwick–Edinburgh Mental Wellbeing Scale (WEMWBS; Clarke et al., 2011; Ringdal et al., 2018; Tennant et al., 2007), Hopkins symptom checklist 10 (HSCL-10; Finbråten et al., 2021; Strand et al., 2003) and Hopkins symptom checklist 5 (HSCL-5; Strand et al., 2003; Tambs and Moum, 1993).

Although females generally experience worse mental health than males during adolescence—presumably in the present samples as well—we do not make any specific hypotheses regarding the mean level differences of the latent factors across sexes. Instead, we adopt an exploratory strategy to test for measurement invariance and specify MIMIC models to investigate the psychometric properties of WEMWBS, HSCL-10, and HSCL-5, across sexes at two important time points in adolescence (school transitions to lower and upper secondary school—grades 8 and 11 in Norway). Our research questions are as follows:

1. Are the instruments WEMWBS, HSCL-10, and HSCL-5, psychometrically equivalent in terms of group-based measurement invariance across sexes in adolescence?
2. Are there disparities across sexes in the observed indicators of the latent constructs WEMWBS, HSCL-10, and HSCL-5, in adolescence?

## Methods

### Sample and procedure

This study includes two samples of students in lower and upper secondary schools, respectively. The lower secondary school sample comprises grade-8 students, and data were collected in the spring of 2021. The data from upper secondary school was collected in 2017, during the spring term of the first year of upper secondary education (grade 11). The samples in this study are from large research projects described in more detail in the next section. For transparency, both projects followed the ethical standards for good practice established by the Norwegian Data Protection Authority and have been approved by the previously called Norwegian Centre for Research Data (NSD), now named Norwegian Agency for Shared Services in Education and Research (Sikt).

The sample of grade 8 students comes from a large research project called Resilient, an RCT with a universal school-based social and emotional learning (SEL) intervention to increase students’ motivation and mental health (Rege et al., 2025).^1^ This In total, 2,146 grade-8 students were invited to participate in the project; among these, 1,968 (91.7%) accepted the invitation, with their parents or guardians signing the consent on their behalf due to their young age at the time (13–14 years). The sample comprised 87 classes from 25 lower secondary schools across three municipalities in the Southwest of Norway. The number of participants in the data collection in March 2021 was 1,608 (49% females; 51% boys).

The sample from upper secondary school (grade 11) is from the COMPLETE project (Larsen et al., 2018), a school-based intervention project designed to improve the completion rate in upper secondary education. Seventeen upper secondary schools across four municipalities in Western and Northern Norway participated in the study. All students at these schools (*n* = 2,942) were invited to complete a questionnaire, and 2,327 students (79%) did so during data collection in March 2017. At this time point, the students were in their first year of upper secondary school. The participants’ ages ranged from 16 to 25 years, and most students were 16 or 17 on the measurement occasion. Because all students were above 16 years of age, they provided their consent to participate in the study. There were 1,282 (55.1%) males and 1,045 (44.9%) females in the upper secondary sample.

### Measures

The Warwick–Edinburgh Mental Wellbeing Scale (WEMWBS: Clarke et al., 2011; Ringdal et al., 2018; Tennant et al., 2007) was used to assess students’ mental wellbeing in both samples (grades 8 and 11). The scale consists of 14 items (see Table 1). Participants were asked to rate how often they had “felt and thought like this” over the last 14 days on a Likert scale ranging from 1 (not at all) to 5 (all the time).

**Table 1:** Overview of items for WEMWBS, HSCL-10, and HSCL-5, respectively, with indicators of the theorized conceptualizations.

WEMWBS		HSCL-10	HSCL-5	
Items	Theorized content at the item level	Items	Items (original number)	Theorized content at the item level
		Overlap for HSCL-10 and HSCL-5		
1. I’ve been feeling optimistic about the future	Eudaimonic	1. Suddenly scared for no reason		Anxiety
2. I’ve been feeling useful	Eudaimonic	2. Feeling fearful	1. Feeling fearful	Anxiety
3. I’ve been feeling relaxed	Hedonic	3. Faintness, dizziness, or weakness		Anxiety
4. I’ve been feeling interested in other people	Eudaimonic	4. Feeling tense or keyed up		Anxiety
5. I’ve had energy to spare	Hedonic	5. Blaming yourself for things		Depression
6. I’ve been dealing with problems well	Eudaimonic	6. Difficulty in falling asleep or staying asleep		Depression
7. I’ve been thinking clearly	Eudaimonic	7. Feeling blue	4. Feeling blue	Depression
8. I’ve been feeling good about myself	Hedonic	8. Feeling of worthlessness		Depression
9. I’ve been feeling close to other people	Eudaimonic	9. Feeling everything is an effort		Depression
10. I’ve been feeling confident	Hedonic	10. Feeling hopeless about the future	3. Feeling hopeless about the future	Depression
11. I’ve been able to make up my own mind about things	Hedonic		4. Nervousness or shakiness inside	Anxiety
12. I’ve been feeling loved	Hedonic		5. Worrying too much about things	Anxiety
13. I’ve been interested in new things	Eudaimonic			
14. I’ve been feeling cheerful	Hedonic			

The Hopkins symptom checklist 10 (HSCL-10; Finbråten et al., 2021; Strand et al., 2003) and Hopkins symptom checklist 5 (HSCL-5; Strand et al., 2003; Tambs and Moum, 1993) were used to assess psychological distress among adolescent students in the, respectively, lower and upper secondary school samples (see Table 1 for an overview). The different versions of the HSCL were administered to the two samples based solely on the questionnaire length (Strand et al., 2003). The 5-item version was administered within the project with the more extensive survey design (the upper secondary school sample), and the 10-item version was administered to the other project (the lower secondary school sample). Adolescents were asked if they, during the previous week, had any symptoms of anxiety and depression. In HSCL-10, four items indicate anxiety (items 1–4), and six items indicate symptoms of depression (items 5–10). HSCL-5 consists of three anxiety items (items 1, 2, and 5) and two depression indicators (items 3 and 4). HSCL-10 and HSCL-5 have four response options for each indicator (1–4): “Not at all”; “A little”; “Quite a bit”; and “Extremely.” Higher scores on the scales indicate higher levels of psychological distress, and a suggested cut-off used to predict the presence of mental disorders and or/belonging to a high-risk group concerning mental health is set to >1.85 in the HSCL-10 and >2.00 in the HSCL-5 (Strand et al., 2003). The scales were previously validated among younger adolescents in the Norwegian educational context and showed acceptable reliability (e.g., Gjerde et al., 2011; Skrove et al., 2013; Strand et al., 2003).

Biological sex (i.e., sex assigned at birth) information was obtained from registry data in both samples. Males were coded as 0, and females were coded as 1.

### Analytic strategy

First, we present descriptive statistics for the study variables, including mean, standard deviation, skewness, and kurtosis for each sample (Table 2). Second, measurement invariance across groups (sex) for each variable within each sample was tested (Tables 3, 4). Multigroup measurement invariance tests were performed to assess the overall equivalence of study measures across groups (sex). This was done by establishing a latent factor structure across groups (i.e., configural invariance) with no constraints on the indicators. Next, to compare variance and covariance across sexes, we constrained factor loadings to be equal across groups (i.e., metric/weak invariance). Finally, to compare latent trait scores across sexes, we added equality constraints to the corresponding indicator intercepts across groups (i.e., scalar/strong invariance). We considered the model fit cut-offs between each model using the recommendations of Chen (2007). Acceptable changes between invariance levels were as follows: change in the Comparative Fit Index (ΔCFI) ≤ 0.010, change in the Root Mean Square Error of Approximation (ΔRMSEA) ≤ 0.015, and change in the Standardized Root Mean Square Residual (ΔSRMR) ≤ 0.030.

**Table 2:** Descriptive statistics of the study variables.

Construct	n	*ω*	Min–Max	Skew	Kurtosis	Boys		Girls	
						Mean	SD	Mean	SD
Lower secondary sample (grade 8)									
HSCL-10	1,541	0.93	1–4	1.05	0.43	1.61	0.68	2.00	0.76
WEMWBS	1,551	0.94	1–5	0.61	0.66	3.74	0.74	3.54	0.77
Upper secondary sample (grade 11)									
HSCL-5	2,232	0.90	1–4	0.89	−0.10	1.60	0.70	2.20	0.85
WEMWBS	2,185	0.96	1–5	−0.48	0.30	3.61	0.86	3.35	0.82

ω = omega reliability, SD = standard deviation, Min–Max = minimum–maximum.

**Table 3:** Measurement invariance of HSCL-10 and WEMWBS in grade 8 across sexes.

	*χ* ^2^	*df*	RMSEA [90% CI]	CFI	SRMR	ΔRMSEA	ΔCFI	ΔSRMR
HSCL-10								
Configural	246.345	68	0.058 [0.051, 0.066]	0.972	0.027			
Metric	265.637	77	0.056 [0.049, 0.064]	0.971	0.032	0.002	0.001	0.005
**Scalar** ^a^	**294.681**	**86**	**0.056 [0.049, 0.063]**	**0.966**	**0.036**	**0.000**	**0.005**	**0.004**
WEMWBS								
Configural	776.304	152	0.073 [0.068, 0.078]	0.929	0.038			
Metric	806.127	165	0.071 [0.066, 0.076]	0.928	0.044	0.002	0.001	0.006
**Scalar** ^b^	**883.885**	**175**	**0.072 [0.068, 0.077]**	**0.920**	**0.048**	**0.001**	**0.008**	**0.004**

^a^One intercept constraint was released for model fit. ^b^Three intercept constraints released for model fit.

The accepted levels of invariance are enhanced in bold.

**Table 4:** Measurement invariance of HSCL-5 and WEMWBS in grade 11 across sexes.

	*χ* ^2^	*df*	RMSEA [90%CI]	CFI	SRMR	ΔRMSEA	ΔCFI	ΔSRMR
HSCL-5								
Configural^a^	21.125	8	0.038 [0.019, 0.059]	0.996	0.010			
Metric^b^	31.980	11	0.041 [0.025, 0.058]	0.994	0.024	0.003	0.002	0.014
Scalar	**55.163**	**15**	**0.049 [0.036, 0.063]**	**0.989**	**0.036**	**0.008**	**0.005**	**0.012**
WEMWBS								
Configural^c^	1,113.883	150	0.077 [0.073, 0.081]	0.935	0.034			
Metric	1,173.051	163	0.075 [0.071, 0.079]	0.932	0.041	0.002	0.003	0.007
Scalar^d^	**1,329.273**	**173**	**0.078 [0.074, 0.082]**	**0.922**	**0.048**	**0.003**	**0.010**	**0.007**

^a^One error covariance was specified for model fit. ^b^One factor loading constraint released for model fit. ^c^Two error covariances were specified for model fit. ^d^Three intercept constraints released for model fit.

The accepted levels of invariance are enhanced in bold.

Third, several structural equation models (SEM) for multiple indicator multiple causes (MIMIC) were used in the analytic work to identify differential item functioning across sexes for psychological distress (HSCL-10 and HSCL-5) and subjective wellbeing (WEMWBS), respectively. Differential item functioning (DIF) occurs when an item on a test or questionnaire has different measurement properties for one group of people compared with another, irrespective of mean differences in the construct (Woods et al., 2009). In estimating the MIMIC model, sex was used as the observed variable and predictor of the latent variable. Thus, a confirmatory factorial approach (CFA) was used to fit the data to measurement models. Group differences were examined for each indicator while holding the other indicators as anchors (i.e., constrained to be equal) across groups. Finally, final models were specified wherein all indicators that were significantly different across sexes were freely estimated, with the other items constrained to be equal.

The analysis was conducted using Mplus version 8.11 software. For each SEM model, we used the recommended cut-offs of CFI > 0.90, RMSEA < 0.08, and SRMR < 0.08, indicating acceptable model fit (Chen, 2007). Since both samples in the present study comprised students clustered within classrooms, we examined the clusters in our data and their impact on the study’s variables. The school- and classroom-level ICCs for mental wellbeing and psychological distress were lower than *r* < 0.05, indicating that schools and classrooms were not necessarily more similar than dissimilar concerning the study’s constructs. However, as our samples were clustered, we used classrooms as a cluster variable and the “type = complex” option for estimation. Missing data were addressed using the full maximum likelihood estimator, assuming that the missing data were missing at random. This approach aimed to maximize the potential of all available information from the data.

## Results

### Descriptive statistics

All factors in both samples achieved acceptable reliability (*ω* > 0.90), and skewness and kurtosis were within acceptable distribution ranges. See Table 2 for details.

### Measurement invariance across sexes

In the grade-8 sample, WEMWBS and HSCL-10 achieved partial scalar measurement invariance across sexes. See Table 3 for details. In the WEMWBS, the items 4, 9, and 12 intercepts were allowed to be freely estimated across groups to achieve an acceptable model fit. The intercept constraint of item 6 in the HSCL-10 was removed to achieve partial scalar invariance across sexes.

Please see Table 4 for details on the measurement invariance across sexes in the grade-11 sample. In the HSCL-5, one error covariance was specified based on the recommendations of modification indices to achieve an acceptable configural model fit. The error covariance was between item 1 and item 2. To establish metric (weak) invariance, the factor loading constraint of item 1 across sexes was removed. Two error covariances were specified in the WEMWBS in the same sample to achieve an acceptable configural model fit. The first error covariance was between items 8 and 10, and the second was between items 10 and 11. Finally, three item intercept constraints were removed to achieve partial scalar invariance across sexes. The item intercepts of items 3, 4, and 12 were freely estimated across groups.

Common in both the grade-8 and grade-11 samples were the discrepancies between sexes on the intercept of the WMWBS items “I’ve been feeling interested in other people” and “I’ve been feeling loved.”

### Multiple indicators multiple causes

**Figure 1:**
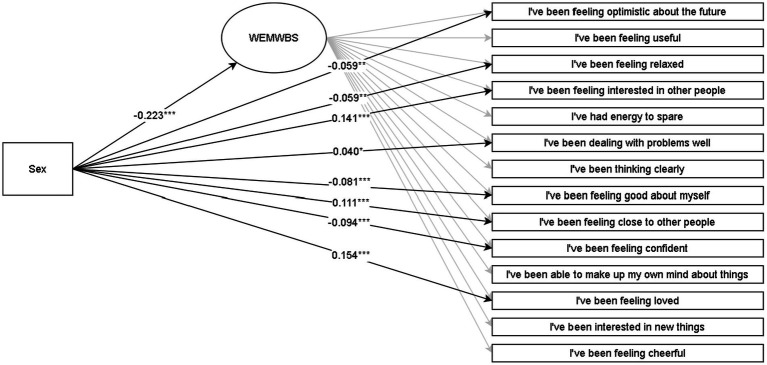
Multiple indicators multiple causes (MIMIC) SEM model for WEMWBS in the grade-8 sample. *p < 0.05; **p < 0.01; ***p < 0.001. The coefficients are Y standardized. Males are coded as 0, and females are coded as 1.

**Figure 2:**
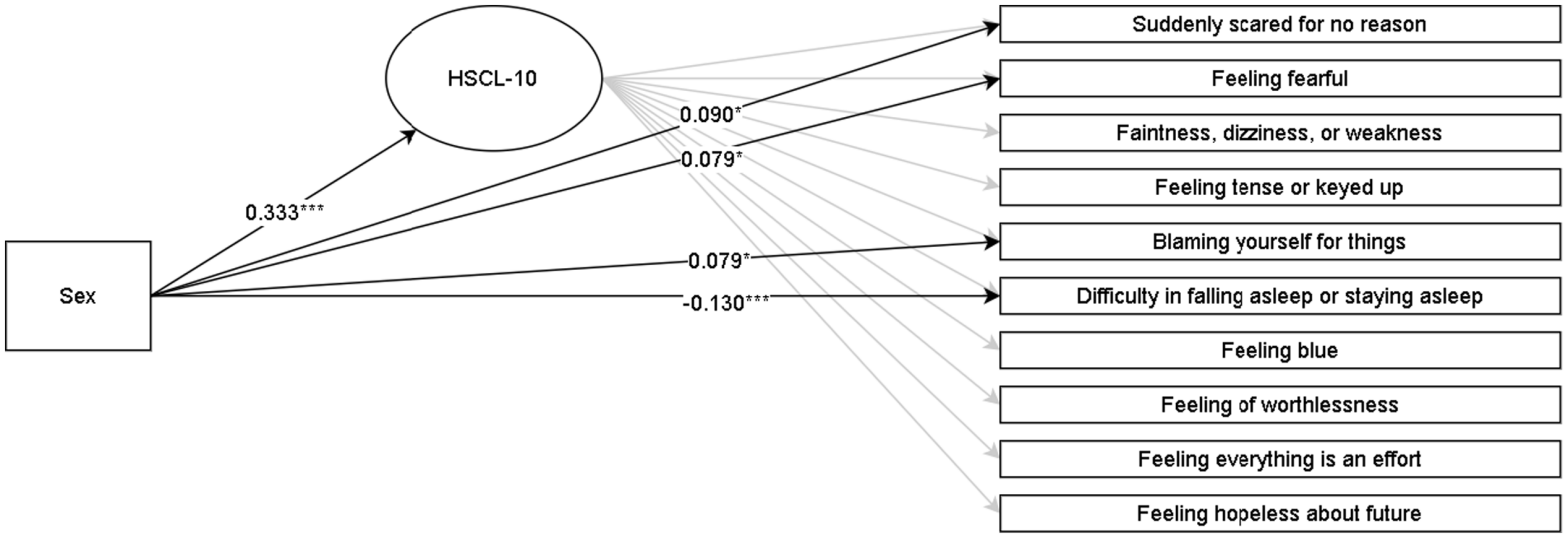
Multiple indicators multiple causes (MIMIC) SEM model for HSCL-10 in the grade-8 sample. *p < 0.05; **p < 0.01; ***p < 0.001. The coefficients are Y standardized. Males are coded as 0, and females are coded as 1.

**Figure 3:**
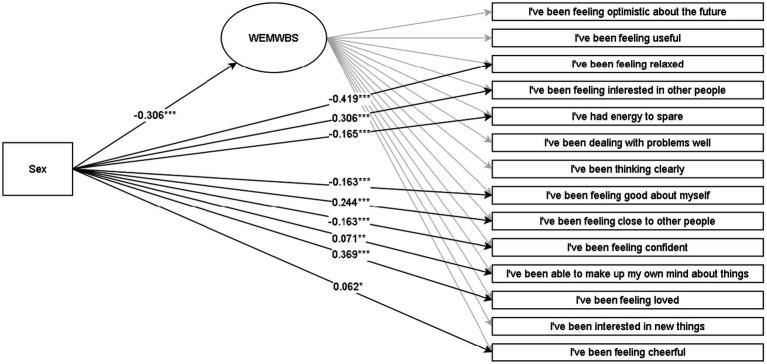
Multiple indicators multiple causes (MIMIC) SEM model for WEMWBS in the grade-11 sample. *p < 0.05, **p < 0.01, ***p < 0.001. The coefficients are Y standardized. Males are coded as 0, and females are coded as 1.

**Figure 4:**
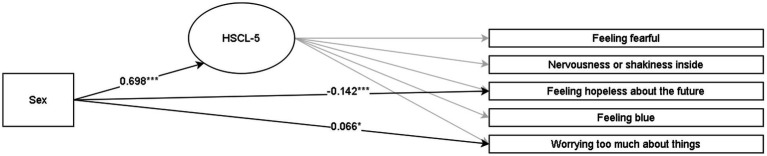
Multiple indicators multiple causes (MIMIC) SEM model for HSCL-5 in the grade-11 sample. *p < 0.05, **p < 0.01, ***p < 0.001. The coefficients are Y standardized. Males are coded as 0, and females are coded as 1.

Discussed below are the visual representations of the results of the MIMIC structural equation models (see Figures 1–4 and Appendix A for more details). All MIMIC models in both samples achieved acceptable model fit (RMSEA < 0.08, CFI > 0.90, and SRMR < 0.08).

In line with RQX, the overall association of sex with mental wellbeing in grades 8 and 11 suggests that males reported higher wellbeing than females. In contrast, allowing sex to be associated with all observed indicators in addition to the latent variable broadened these nuances at the indicator level. This matter was also supported statistically by comparing the overall model with the model where non-significant associations of sex with observed indicators were constrained to zero, in which the latter yielded a significantly better fit in the grade 8 (Δ*χ*^2^ = 246.51 [Δ8], *p* < 0.001) and grade 11 (Δ*χ*^2^ = 575.77[Δ9], *p* < 0.001) samples. In both lower and upper secondary samples, results at the observed indicator level suggest that males, to a greater extent, reported aspects related more to hedonic feelings (feeling relaxed and good about oneself). Females tended to report higher levels regarding eudaimonic matters of meaningfulness and social relations (interest in others, closeness to others, and feeling loved).

Concerning symptoms of anxiety and depression (HSCL-10 and HSCL-5), females reported higher overall levels than males in both grade 8 and 11. The HSCL-10 model in the grade-8 sample with all coefficients constrained to zero yielded a worse fit than the model with the four significant regression coefficients freely estimated (Δ*χ*^2^ = 24.93 [Δ4], *p* < 0.01). Similarly, the HSCL-5 model (grade-11 sample) with all indicator coefficients constrained to zero had a significantly worse fit than the model wherein the two significant regression coefficients were freely estimated (Δ*χ*^2^ = 17.03 [Δ2], *p* < 0.001). The HSCL-10 and HSCL-5 results indicated that females reported higher scores on the items specific to anxiety (feeling fearful and scared, blaming oneself for things, and worrying too much) across the lower and upper secondary school levels. In contrast, males reported higher scores on the indicators that are specific to depression (difficulty in falling or staying asleep and feeling hopelessness about the future).

## Discussion

The primary aim of this study was to examine the potential sex differences in widely used adolescent mental health instruments. By utilizing established and validated measures that assess mental wellbeing (WEMWBS) and psychological distress (HSCL-10 and HSCL-5), we aimed to gain a more comprehensive understanding that can enhance knowledge, improve measurement development, and inform intervention strategies to support adolescent mental health effectively. We discuss the findings below, using an ecological relational perspective to highlight the unique aspects of adolescent mental health.

### Equivalence in mental wellbeing and psychological distress across sexes in adolescence

In response to the first research question of this study, the findings indicate that the measures assessing mental wellbeing and psychological distress only partially achieve invariance at the latent factor level. In both samples (grades 8 and 11), the results for mental wellbeing revealed a discrepancy in item 4, which pertains to feelings of being loved. This item reflects the hedonic aspect of mental wellbeing, emphasizing the importance of maximizing pleasure and minimizing pain (Deci and Ryan, 2008). A similar discrepancy was found for both samples in item 12, which addresses the eudaimonic aspect of mental wellbeing, specifically the statement, “I’ve been interested in other people.” These discrepancies support the existence of potential differences at the item level and thus contradict previous research that has found consistency across sexes (Putnick and Bornstein, 2016). In other words, the sex discrepancies in items 4 and 12 of the WMWBS might indicate a dissimilar contribution of indicators to their latent construct or a mean difference in indicators that are not captured by the latent construct. Thus, we suggest that researchers investigate these discrepancies further and be cautious about the potential measurement bias across sexes that these items pose in the WMWBS.

Regarding psychological distress, the grade-8 sample did not achieve full factorial measurement invariance for the item related to sleep problems. The non-invariance of item 6 was unsurprising as the finding supports earlier studies suggesting differences in how females and males interpret sleep issues (Finbråten et al., 2021; Kleppang and Hagquist, 2016). Because this item seems to pose recurring measurement bias across sexes, it might need to be reevaluated as an indicator of the latent construct symptoms of anxiety and depression in the HSCL-10. Hence, the item ‘difficulty falling asleep or staying asleep’ might be reconsidered when researchers are administering the HSCL-10 measure. Measurement bias may persist even when gender and/or sex are not of interest.

In the grade-11 sample, item 1, which addresses feelings of fear, also failed to demonstrate factorial equivalence across sex. It is previously indicated that females experience higher levels of fear than males (Ollendick et al., 2002). One explanation for this discrepancy has been linked to gender role orientation, wherein young people are socialized to develop behaviors, traits, and skills associated with their sex assigned at birth (Ollendick et al., 1995). Classic gender role theory argues that expressing fear is more aligned with the feminine gender role, while it is expected that people with a masculine gender role are more courageous and brave (Bem, 1981). In other words, items measuring fear might need to be further investigated as indicators of psychological distress, and for the potential bias they introduce to the instruments. It might be beneficial to investigate such items in relation to gender typical behaviors, traits, and skills to further untangle the presently observed sex discrepancy and the effect it might have on the latent construct of psychological distress.

### Sex disparities of WEMWBS and HSCL-10/HSCL-5 in adolescence

Regarding the second research question of this study, the findings show that males report overall higher levels than females on the latent factor of wellbeing in both samples. Considering that younger male adolescents are found to report higher wellbeing compared to females, which diminishes over this period (Gestsdottir et al., 2015), our findings showed an average greater wellbeing for males than females in early as well as in later adolescence.

However, findings of the differential item functioning for wellbeing suggest that being male is associated with hedonic indicators such as feeling good about oneself, feeling confident and relaxed, and having energy to spare. Being female was in association with eudaimonic indicators such as feeling interested in, close to, and loved by other people, dealing with problems well, and making up their minds about things. These findings might partly be explained by sex differences in the motivations people have for pursuing wellbeing. For instance, LeFebvre and Huta (2021) found that eudaimonic motivation (seeking meaning, authenticity, excellence, and growth) increased for adult females until their 30s, while males in their 20s scored significantly higher in hedonic pleasure motivation (seeking pleasure, enjoyment, and fun) than females.

The significant association between being male and feeling optimistic about the future was only present in the grade-8 sample, not in the grade-11 sample. Similarly, the indicator of feeling hopeless about the future was only significantly associated with being male in the grade-11 sample, not grade 8. These findings may indicate that being male is related to optimism in early adolescence and hopelessness in later adolescence. It is not known if the changes in these effects are due to the general loss of optimism as children grow older (Habicht et al., 2022) or if males are at risk of experiencing a negative trajectory of hopelessness from adolescence to young adulthood (Langhinrichsen-Rohling et al., 1998). A further investigation of male hopelessness and loss of optimism across adolescence might provide important information pertaining to the paradoxical sex disparities in suicide.

While being female was associated with indicators of anxiety in the grade-8 and grade-11 samples, being male was linked to difficulties falling or staying asleep in grade 8 and feelings of hopelessness in grade 11, both of which are indicators of depressive symptoms. Because males tend to express negative emotions with increased substance use and externalizing behaviors such as aggression and risk-taking (e.g., Cochran and Rabinowitz, 2000), the current measurement tools to identify psychological distress might be biased towards females. It is possible that excluding externalizing behaviors in measurement scales might contribute to the skewed perspective of sex differences in mental health because the instruments inadequately identify negative affective conditions in males (e.g., Cochran and Rabinowitz, 2000, 2003; Rochlen et al., 2010).

In summary, female distress primarily relates to anxiety symptoms, whereas wellbeing concerns eudaimonic factors, which may contribute to resilience against depressive symptoms, aligning with the findings of (LeFebvre and Huta, 2021). By contrast, males tend to score higher on indicators of depressive symptoms and on hedonic aspects of wellbeing (such as feeling happy), suggesting a negative mental health development that is likely to involve complex interactions. Indeed, our findings suggest that using the same indicators for both sexes regarding wellbeing and psychological distress may have significant consequences, particularly in prevalence reporting and implementation research. Our findings also align with previous research studies in which adolescent male depressive symptoms might be challenging to recognize (Barrigon and Cegla-Schvartzman, 2020). This also supports the notion that we miss pieces of a more complex puzzle regarding adolescent sex disparities in mental health instruments.

## Limitations and future directions

One limitation of the present study was the cross-sectional design. Although we collected data on different age groups, we did not follow the same participants across time. Thus, we make no direct inferences about developmental changes or cause-and-effect relationships throughout adolescence. However, because we included two samples from different age groups at two time points, we can infer trends within and across sexes and age groups during adolescence.

Second, the study’s samples are not nationally representative. Thus, we advise caution in generalizing our results to the entire Norwegian adolescent population or other adolescent populations. Since the two samples represent different age groups (young and older adolescents) at various educational stages, creating contextual distinctions, this should also be taken into account when interpreting the results. Nevertheless, the participants from both projects come from a mix of rural, semi-urban, and urban areas in small, medium, and large schools. Moreover, both projects feature a similar representation of both sexes.

The present study provides new and novel information about sex differences at an indicator level in widely used mental health instruments. However, we acknowledge that more research is needed to achieve the overarching goal of developing fair and unbiased instruments to measure adolescent mental health correctly across different groups (in this case, sexes). We suggest that future studies might (1) apply our analytical approach to longitudinal studies to ascertain the effects found in the present study, (2) include other socio-demographic variables (e.g., socio-economic status, cultural or environmental factors, race, and so on) in similar studies to ours, to untangle further how such factors might influence or confound the association between sex and mental health indicators, and (3) investigate potential mechanisms underlying the sex differences in certain indicators to identify further developmental pathways, precursors, and protective factors in mental health problems across sexes.

## Data Availability

The data analyzed in this study is subject to the following licenses/restrictions: the datasets are owned by the prospective projects described in the manuscript. Requests to access these datasets should be directed to Lene Vestad, lene.vestad@uis.no and Sara Madeleine Kristensen, madeleine.kristensen@korusnord.no.

## Footnotes

^1^Rege, M., Bru, B., Solli, I., Thijssen, M., Tharaldren, K., Vestad, L., et al. (2025). The impact of teaching coping skills in schools on youth mental health and academic achievement: evidence from a field experiment. Manuscript in preparation.

## Appendix A

### Overview of indicators showing potential bias across sexes in WEMWBS and HSCL-5/10

**Table A2:** Indicators with significantly higher levels for males across grade levels

Instrument indicators	Grade 8	Grade 11
WEMWBS		
I’ve been feeling optimistic about the future	X	
**I’ve been feeling relaxed**	X	X
I’ve had energy to spare		X
**I’ve been feeling good about myself**	X	X
I’ve been feeling confident	X	
HSCL-10		
Difficulty falling asleep or staying asleep	X	
HSCL-5		
Feeling hopeless about the future		X

Indicators in bold showed potential bias in both samples (grades 8 and 11).

**Table A1:** Indicators with significantly higher levels for females across grade levels.

Instrument indicators	Grade 8	Grade 11
WEMWBS		
**I’ve been feeling interested in other people**	X	X
I’ve been dealing with problems well	X	
**I’ve been feeling close to other people**	X	X
I’ve been able to make up my own mind about things		X
**I’ve been feeling loved**	X	X
I’ve been feeling cheerful		X
HSCL-10		
Suddenly scared for no reason	X	
Feeling fearful	X	
Blaming yourself for things	X	
HSCL-5		
Worrying too much about things		X

Indicators in bold showed potential bias in both samples (grades 8 and 11).
